# The value of diabetes technology enabled coaching (DTEC) to support remission evaluation of medical interventions in T2D: Patient and health coach perspectives

**DOI:** 10.1371/journal.pdig.0000701

**Published:** 2025-01-09

**Authors:** Madison Taylor, Denise Ng, Kaylen J. Pfisterer, Joseph A. Cafazzo, Diana Sherifali

**Affiliations:** 1 Centre for Digital Therapeutics, Toronto General Hospital Research Institute, University Health Network, Toronto, Ontario, Canada; 2 Systems Design Engineering, University of Waterloo, Waterloo, Ontario, Canada; 3 Institute of Health Policy, Management and Evaluation, Dalla Lana School of Public Health, University of Toronto, Toronto, Ontario, Canada; 4 Institute of Biomedical Engineering, University of Toronto, Toronto, Ontario, Canada; 5 Department of Computer Science, University of Toronto, Toronto, Ontario, Canada; 6 School of Nursing, McMaster University, Hamilton, Ontario, Canada; 7 Population Health Research Institute, McMaster University, Hamilton, Ontario, Canada; 8 Department of Health Research Methods, Evidence, and Impact, McMaster University, Hamilton, Ontario, Canada; Iran University of Medical Sciences, ISLAMIC REPUBLIC OF IRAN

## Abstract

The multicomponent Remission Evaluation of Medical Interventions in T2D (REMIT) program has shown reduction of hazard of diabetes relapse by 34–43%, but could benefit from improved ability to scale, spread, and sustain it. This study explored, at the conceptualization phase, patient and health coach perspectives on the acceptability, adoption, feasibility, and appropriateness of a digital REMIT adaptation (diabetes technology enabled coaching (DTEC)). Twelve semi-structured interviews were conducted with patients (n = 6) and health coaches (n = 6) to explore their experiences with the REMIT study, opportunities for virtualisation, and a cognitive walkthrough of solution concepts. Transcripts were analyzed both inductively and deductively to allow for organic themes to emerge and to position these themes around the constructs of acceptability, adoption, feasibility, and appropriateness while allowing new codes to emerge for discussion. Participants saw value in DTEC as: an opportunity to facilitate and extend REMIT support; a convenient, efficient, and scalable concept (acceptability); having potential to motivate through connecting behaviours to outcomes (adoption); an opportunity for lower-effort demands for sustained use (feasibility). Participants also highlighted important considerations to ensure DTEC could provide compassionate insights and support automated data entry (appropriateness). Several considerations regarding equitable access were raised and warrant further consideration including: provision of technology, training to support technology literacy, and the opportunity for DTEC to support and improve health literacy. As such, DTEC may act as a moderator that can enhance or diminish access which affects who can benefit. Provided equity considerations are addressed, DTEC has the potential to address previous shortcomings of the conventional REMIT program.

## Author summary

We interviewed patients and health coaches on how technology *plus* health coaches could improve the management of people living with type 2 diabetes. Participants named convenience and efficiency as benefits. They noted how adding technology like an app could help patients draw deeper connections between foods eaten and their impact on their body. However, participants also shared important considerations. One, entering data needs to be easy. Two, was about fairness—both the technology and appropriate training need to be provided. Our main take-away is while technology plus health coaching may add value, careful thought around operationalization is needed.

## Introduction

Currently, type 2 diabetes (T2D) management involves lifestyle modification and a step-wise addition of glucose-lowering medication in response to rising glucose levels and failing therapeutic regimens [1]. This approach leads to an inconvenience of frequent self monitoring of blood glucose (SMBG), clinical appointments to monitor the effectiveness and side effects of therapies, coupled with chronic and expensive polypharmacy which is associated with increased risk of adverse effects, non-adherence to medications, and poor quality of life [2–7]. Evidence that T2D can be completely or partially reversed is now challenging the traditional approach to its management [8–20]. Diabetes remission (defined as a return to glycemic levels below the threshold of diabetes diagnosis without the ongoing need for glucose-lowering medications) has been achieved with bariatric surgery and with the implementation of a very low-calorie diet for a limited time [21]. However, bariatric surgery is not accessible or acceptable to many patients and not scalable for primary care, where the vast majority of diabetes care is delivered in Canada [14–20], and non-surgical approaches to achieving remission are being studied with randomized trials or other research designs [22–26].

The Remission Evaluation of Medical Interventions in T2D (REMIT) program comprises 4 pan-Canadian multicentre (8 sites) trials, and one ongoing trial of the paradigm of a 4-month multimodal intervention that comprises remission-induction period, followed by passive follow-up to detect relapse [27–30]. The REMIT program has tested a 4 month induction period using frequent health coaching focused on self-efficacy, weight reduction and increased physical activity, and in addition to metformin, glargine insulin and either: a) dapagliflozin (SGLT2 inhibitor); b) sitagliptin (DPP4 inhibitor): or c) lixisenatide (GLP-1 receptor agonist) [27–29,31]. The ongoing trial comprises a 4-month induction period for which the drug components is metformin, degludec insulin and liraglutide. This intervention reflected the paradigm of reducing pancreatic beta-cell work and increasing insulin sensitivity to reverse glucotoxicity and partially or completely restore pancreatic endocrine function [22–26,32]. All 4 trials recruited patients of various sociodemographic and ethnic backgrounds from across Canada, diagnosed with T2D (<5 years) [27–30]. Participants were assigned to either usual care or the remission program for the duration of the remission-induction period. At the end of this period, participants with a glycated hemoglobin (A1C) <6.0% (42 mmol/mol) or <6.5% (48 mmol/mol) off diabetes medications since 12 weeks after randomization [29]. The 4 trials reported that the intervention group reduced the hazard of diabetes relapse by 34–43%, with 14–23% remaining in the remission intervention group and 8–11% in the control group at 1 year [27–30].

Research is needed to test the implementation of a remission approach in real-world settings, including the use of technology-enabled diabetes remission interfaces, apps and health coaching to scale, spread and sustain this multicomponent intervention in a cost-effective and efficient approach. The ubiquitous use of smartphones by the general population has provided an opportunity to reach diverse populations across geographical regions, deliver sophisticated tools for diabetes self-management through mobile applications (apps), and facilitate digital behaviour change health coaching programs [10]. However, the vast majority of apps are not rooted in evidence and lack foundations in theoretical models [11,12].

This study builds on previous efforts by bringing together expertise in diabetes coaching with digital health. This study represents the conceptualization phase of a new technology for remotely delivering diabetes technology-enabled coaching (DTEC), and how DTEC may be positioned to improve outcomes at the patient, clinical and healthcare system level. DTEC comprises diabetes coaching, along with healthy behaviour data analytics, goal monitoring and attainment reporting, and online educational resources. The purpose of this study was to understand whether a digital adaptation of the REMIT intervention would be palatable, and identify opportunities both to improve the patient and provider experience with the REMIT as well as how technology might enable a more scalable experience. Perspectives captured through this work include individuals living with T2D who have completed a previous REMIT trial, and health coaches of the REMIT study program. Specifically, this study explored at the conceptualization phase, patient and health coaches perspectives of the acceptability, adoption, feasibility, and appropriateness of a DTEC to support the future implementation of a multimodal remission intervention.

## Results

**Fig 1:**
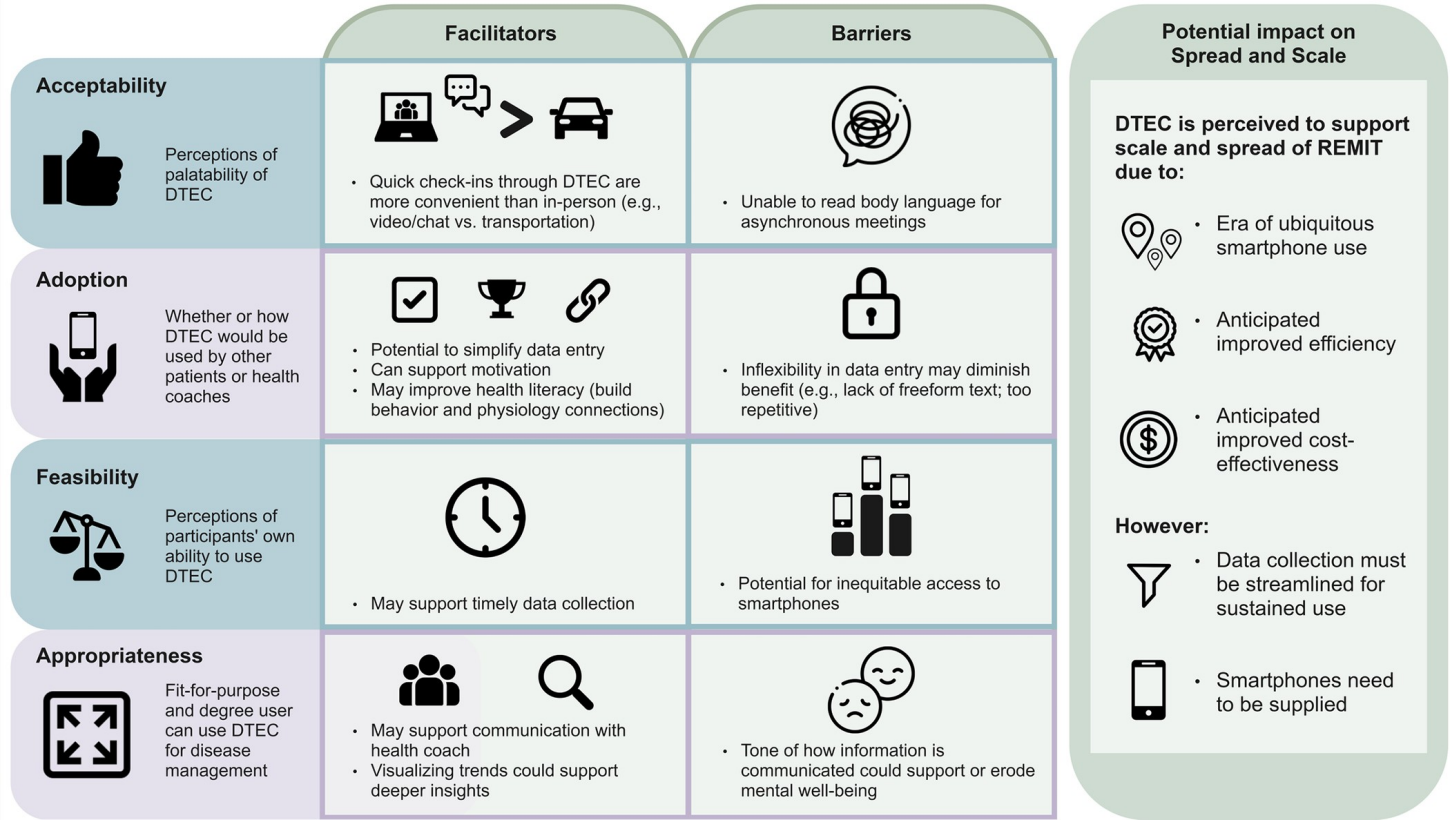
Summary of patient and health coach perceptions of DTEC’s acceptability, adoption, feasibility, and appropriateness, related barriers and facilitators, and potential impact on the spread and scale of REMIT.

Results provide an overview of sample demographics, health coach experiences with REMIT as well as barriers, and opportunities for virtualized care to support the implementation of the REMIT intervention as summarized visually in Fig 1. Results then explore the value participants see in DTEC exploring acceptability, adoption, feasibility, and appropriateness within the context of supporting the scale and spread of the REMIT intervention. We conclude results with a summary of participant cognitive-walkthrough feedback to support health knowledge disparities, accessible design, and language use.

### Sample demographics

Our remission health coaches interviewed came from a variety of backgrounds such as, nursing, dietetics, and general health sciences (i.e., research coordinators). Our sample had between 2 to over 20 years of experience as diabetes educators with typical rosters of clients between 10 to 60 at a given time. Health coaches described their clients as typically overweight, and between 40 to 70 years of age; the majority were 50+. Our sample of patients had a mean age of 58.6 (SD 10.7) years of age, 67% (4/6) identified as female, with at least high school education (3/6 with a graduate or other advanced degree), and had been diagnosed with diabetes between 3 and 6 years. All patients had regular access to technology (e.g., a combination of iPads, computers, smart phones) in use between 1 and 4+ hours per day with a mode of technology use of 3–4 hours per day (3/6). Patients reported 33% (2/6) were actively using diabetes management software with 67% (4/6) using a fitness tracker. All patients reported knowing what Bluetooth is and how to use it with 83% (5/6) being comfortable with their skills to use any app. Respectively, 33% (2/6) and 67% (4/6) of patients reported being comfortable, and very comfortable using technology.

### Themes

#### DTEC: An opportunity to facilitate and extend REMIT support

Patients who experienced the REMIT trial experienced positive results such as weight loss without feeling starved, maintaining sugar levels after the trial ended, or reduced medications. One patient shared, *“At the conclusion of the REMIT trial*, *I mean I’d been drug free*, *you know for a year*. *That part was good and it was satisfying to be part of a study that actually showed benefit*.*”* However, patients often wished they could continue to benefit from the intervention. Some noted they felt the intervention was unsuccessful because the results weren’t maintained after the trial. *“Eventually*… *my blood sugar crept up again*, *and then I was back on metformin*. *During the REMIT protocol I was not on metformin*.*”* Others did not hit the targets within the span of the trial but still felt there was value, like this patient who shared, *“I think I left [REMIT] with definitely a better grasp and I keep my numbers at a more suitable level*… *I haven’t had an A1C over the 6*.*4*… *in*… *two years now*.*”*

DTEC may provide a mechanism for extending REMIT support during and after trial completion in a scalable and spreadable way. One patient shared, *“I wouldn’t say I have any certainly negative concerns about the transition out of [REMIT]*… *Except then it was over*.*”* Health coaches and patients shared the challenge of transitioning from having high-touch interactions to no further support. Clients also felt alone, without support, and with a loss of accountability and motivation. They wished for a *“mechanism where you can [give] more of a daily feedback [for] someone*.*”* Health Coaches shared, *“What I would hear is*, *‘you know I talked to you almost every day and you were part of my life and then all of a sudden you were gone*.*’”* And, *“There has to be some way where when you’re no longer there that they can still continue as efficiently as they were and not feel like they’re alone*.*”*

Participants described the opportunity of DTEC to support programmatic components of the REMIT intervention to include messaging and self-management education. A health coach suggested messaging to send and respond to quick one liners, *“You can just say*, *‘hey*, *you know what I was wondering about this is*, *is this something I can even think about or I just heard this on the radio is that relevant*?*’”* This is corroborated by Nelson et al who have provided evidence supporting the use of text messaging both to address scale and reduced health literacy disparities [33]. However, our participants suggest taking this further. Participants desired functionality for supporting carb counting and tailored learning opportunities. For example, a health coach noted, *“There’s so much information [available on the internet and other sources] that they don’t know how to relate it to themselves and personalize it”*. Specific examples of tailoring education included passive methods like curating links to reliable information sources (health coach suggestion), to interactive self-management support to motivate and engage patients through actionable next-steps.

“*Like if you had a glucose reading after like 2 hours after breakfast and [the app says], ‘Oh your glucose reading is a little bit on the higher end. These are the things that you can do next at your next meal to kind of make it lower.’… so a little bit more interactive to… self-guide the user.”–Patient*

#### Acceptability: DTEC is convenient, efficient and scalable

Acceptability was defined as participants’ perceptions of the palatability for DTEC and may affect the scalability and spreadability of DTEC. Health coaches noted in-person meetings were beneficial, especially for reading body language to help them meet clients where they are at. However, several mentioned that in-person can be inaccessible or infeasible, as previously done in the REMIT trials. A health coach shared, *“Asking someone to come in for a 15-minute appointment regularly is a lot for the patient in terms of paying for parking and taking time away from their day and commuting to the hospital*.*”* A patient highlighted, *“[The DTEC prototype] seems to be maybe a quicker method and it’s right at your fingertips*.*”* This general perceived value is further expanded upon through the perceived acceptability, adoption, feasibility and appropriateness of DTEC by health coaches and patients.

Participants noted DTEC would not only support convenience but suspected it would also improve efficiency and be more cost-effective for scaling REMIT. A health coach shared, *“It would allow you not to have to skip appointments or do it over the phone rather than do it in person*. *It also gives you more flexibility [regarding access in remote regions]”*. Another noted, *“We need to figure out how do we take this approach that we’ve been working on and researching for all these years and roll it out to the population in a way that is cost efficient*.*”*

Patients shared enthusiasm for the concept and perceived it to support diabetes management as a means to scale and spread the REMIT intervention. One patient stated, *“I think it’s a great idea*, *I really like the idea of this app*. *I think it’s going to help a lot of people*.*”* Another patient shared,

“*Well, I mean the use of smartphones is the way of the world now, right? So I think it’s very consistent with our use of technology. Diabetes is a complex topic with a bunch of interacting factors, so something that kind of gathers information guides, reports back, connects you with [other individuals]… It’s a great way to go, actually.”–Patient*

#### Adoption: DTEC may motivate through connecting behaviours to outcomes

Adoption was defined as participants’ perceptions of how other patients or health coaches would use it. Participants highlighted how DTEC would support ease of sharing data with their health care team, and simplify tracking which would support its scale and spread. A patient shared, *“I feel like if it’s all in one place it’s easier*, *it’s less overwhelming*, *and again*, *just quicker*.*”* A health coach noted, *“I would love to try it*…*We can look at the blood sugar*, *visuals in the middle of the conversation*, *which we don’t do on a regular basis because it’s not as practical*.*”*

Participants identified that DTEC may help motivate or build intuitive connections between behaviours and their impact on measurements. This value added may positively impact DTEC’s spreadability through potential higher uptake of technology. Health coaches stated, *“It’s practical for a regular clinic appointment that they could bring those numbers to their physician or diabetes nurse in the future outside of the study period*. *And because it tracks overtime*, *it may be motivating for the patient*.*”* and *“seeing visuals of things*…*It’s motivating ‘Hey*, *I’m almost there*. *I’ve done something*.*’ you know*, *give some feeling*. *That they’re on their way and to achieving their goals*.*”* A patient imagined they were looking at their digital health application which showed their blood sugars for the last two weeks and said, “‘*very well hold on*, *this is what I had the last two weeks*. *Oh my God*, *my blood sugars were up in like the fifteens I gotta stay away from that*. *Let’s try something else*.*’ I think that would be a very positive way of keeping people on the right track*.*”* Another patient noted, *“I kind of like the activity or the minutes or the steps or something like that*. *Because then it’s sort of like*, *oh*, *I only have 1200 steps*. *I better go for a walk*.*”*

#### Feasibility: Opportunity for lower-effort demands for sustained use

Feasibility was defined as participants’ self-perception of their own or others’ ability to use a virtualized version of the REMIT intervention given available time, resources, workflow, and skill considerations. Participants identified the opportunity for added flexibility to improve feasibility and sustained use. They noted that while intensive data collection on a digital health platform would be supportive during the intensive period of the REMIT intervention, especially as the level of manual data entry of every meal, activity and blood glucose reading may be burdensome without automation for sustained use.

One patient shared, *“I would like that ability to take a note that said*…*You know*, *I was at a party and throughout the evening [this] is what I ate*.*”* A health coach reinforced this also, *“From my perspective*, *not being a dietitian*, *I don’t think [tracking every pre and post meal with food photography] would be helpful for me and I think it might be burdensome for the patient*.*”* However, within the research context, the value and feasibility may be higher and more supportive for a digital health platform to facilitate data entry. This may indicate the ability to scale and spread DTEC could depend upon which sphere of application is implemented.

"*So it would definitely improve, I think, just participants’ ability to record data in a timely fashion. I would say there are a good number [of research participants] who are, you know, filling out [diaries] in the 5 to 10 minutes before they show up for their appointment.”—Health Coach*

However, equitable access warrants additional consideration for the feasibility of scaling and spreading DTEC. A health coach noted, *“the patient population that did REMIT*… *they would all have smartphones*, *but my regular patient population*, *not necessarily if they were older*.*”* A patient highlighted that, *“You have to have money to be diabetic [which includes having] a smartphone that’s up to date*.*”* A health coach noted *“we don’t see a lot of low income participants*, *I think*, *largely just because they don’t have the flexibility to participate in those kind of protocols*.*”* This has implications for the format through which DTEC could be expanded; a health coach commented, *“There’s a lot of low income patients that don’t have cell phones and so they have to make sure that it’s all provided to the patients*.*”*

#### Appropriateness: Opportunity to provide compassionate insights and automate data entry

Appropriateness was defined as the perceived fit-for-purpose as well as the degree to which the user can use the digital health tool for their disease management which may influence the ability to scale or spread the tool. The two main take-aways were (1) it matters what is being tracked, and (2) how information is communicated can greatly affect the use of DTEC, including possibly having it fail because of how it may interact to support or erode mental well-being.

Regarding what is tracked, participants felt functionality of a digital health platform would be supportive for self-management and health coaching during the remission protocol. Specifically on the ability to communicate through DTEC, one health coach shared, *“I definitely think that with health coaching*, *it’s a very valuable tool to be able to communicate with the patient*…*through the phone calls or videos or emails*. *And I think it really helps with motivation for people*.*”*

Regarding how information is communicated, several participants commented on the value of visualizing trends. For example, a health coach shared, *“It’s a quick glance and it gives you the information you need to target*.*”* Participants commented on how DTEC could support gaining deeper insights. A health coach shared, *“It’s really helpful to see [trends]*… *we kind of can see before breakfast*, *they don’t have any issues with their blood sugars*. *But maybe before dinner is more problematic*.*”* However, participants also flagged that while self-management data, including meal intake, blood glucose medication, exercise and goals are helpful, care should be taken in how results are presented. For example, one patient noted, *“I feel like weight is a good thing to have in there*, *but I feel like it should be a little bit to the background because if it’s too much to the forefront*, *I think it’s too like ohh I failed*.*”* In line with this, patients identified the need for consideration around impacts of data presentation on mental well-being. For example, an overemphasis on BMI or weight may result in feeling like a failure or prohibiting use. One patient said, *“I just hate to think that maybe anybody who’s using this app for the first time will keep seeing BMI and it [might] turn them off*… *might make them go to extremes*, *yeah*?*”*. Another patient commented, *“I do think there should be some set goals like I don’t want to exclude goals but I just feel like you have to be cautious about it*.*”*

### Supporting equity through health knowledge, accessible design, and language

Cognitive walk-throughs yielded two main take-aways. The need for personalized learning opportunities, and the need for accessible design. Participants suggested that directing education could be achieved through supporting carb counting, and providing tailored learning opportunities. For example, a health coach noted, “There so much information [available on the internet and other sources] that they don’t know how to relate it to themselves and personalize it”. Independently, a patient echoed this,

“First you have to read what you’re eating [on] the box or what you’re eating and decide how many carbs you have. I’m wondering if it would just be an easier way for you to reference that sort of information through the app.”—Patient

Specific examples of tailoring education included curating links to reliable information sources. For example, a health coach suggested, “If you have various links that you may have talked about during, using it if that, if that’s readily available.” A patient also suggested the supportive and motivating nature of a more interactive mode of engagement with DTEC facilitate through actionable next-steps.

“Like if you had a glucose reading after like 2 hours after breakfast and is there any way the app can say, ‘Oh your glucose reading is a little bit on the higher end. These are the things that you can do next at your next meal to kind of make it lower.’ That kind of stuff so a little bit more interactive to kind of signal and kind of like guide self-guide the user up”—Patient

Design considerations are important to review relating to colour choices given the population it is meant to serve who require good contrast for readability. For example, using black and white (high contrast) instead of light blue and white (low contrast). Especially in the context of diabetes where there is an increased risk for cataracts, these design elements are crucial for equitable usability and to support adoption.

“I think you have to look at the population that you’re dealing with, which is obviously going to be a much scenically older group, and some people are very adept and other people art can do the basic stuff.”—Patient

One participant highlighted the requirement of precision of language to avoid confusion that may cause distress. Some examples included, when logging food photos, “after a meal” is too ambiguous about what denotes an appropriate time frame, expectations around how to navigate combining readings from multiple devices (e.g., different scales at the dr’s office vs home), and the appropriateness of including BMI when it has received criticism for its individual applicability and has the potential to be triggering from a mental well-being perspective.

## Discussion

Provided acceptability, feasibility and appropriateness of DTEC, it holds the power to scale and spread remission. At the conceptualization phase described here, participants saw value in DTEC through: anticipated improved efficiency and scaling (acceptability), the perceived ability of DTEC to support motivation and to draw causal inferences between food intake and their body’s response (adoption), the opportunity to streamline DTEC for sustained use (feasibility), as well to provide support during and extend beyond REMIT trials (appropriateness). Based on the perceptions shared by this sample of patients and health coaches, DTEC may be able to address previous barriers to access, enhance accountability, and improve self-efficacy. Our results highlighted DTEC is positioned to support that scale and spread with one main caveat regarding potential barriers due to equitable access.

Interdependent considerations are needed for equitable design to address differential access to resources and health knowledge disparities for enhanced self-management. For example, our results suggest that DTEC may act as a moderator that can enhance or diminish access which affects who can benefit. While a digital application may allow for more flexibility in care provision, equitable access is an important consideration when deploying services that rely on digital tools. Ultimately patients are resource constrained. Making the most supportive choices are not always within reach both from access to healthy foods as well as technological access standpoints and this affects who has the opportunity to benefit from DTEC. These factors contribute to the long-term sustainability of DTEC which is also linked to scalability. Alvarado *et al*. note that scalability issues are the most common barrier in mid-income populations [34]. Complementary to our findings, others have noted the explicit need to address more equitable prescribing practices, stronger support for social determinants of health, the need for care models to support patient preferences, as well as cost [35]. In line with this, provision of technology may be necessary but not sufficient. Enhancing technology literacy may also be required, having been cited as the most common barrier in low-income populations [34].

Supporting sustained behavior change is crucial for experiencing an intervention’s lasting impact. Outside of chronic disease management, chatbots appear to enhance engagement and motivation [36,37], and are positioned to provide personalized feedback [37], facilitate on-demand support [37], as well as gamification [38]. Exploring the use of text-based chatbots through short text messaging (SMS) and web services may close the gap on access to technology, as SMS may be more widely accessible and acceptable to equity deserving populations. Particularly in the age of generative artificial intelligence, tools like chatGPT and intelligent conversational agents serve as assistive technology for those with different levels of abilities [39–41]. More broadly, there is great potential for this technology to assist in information gathering, consolidation, and interpretation which may aid in closing the access gap for individuals with varied levels of both digital and health literacy [36,37]. Within chronic disease management application of these technological advances is accelerating as well. Nelson *et al* showed how AI-augmented health coaching may address gaps on sustained effects of texts alone [33]. A 2023 scoping review on conversational agents (a form of chatbot) reported on the potential for these agents to improve weight-related behaviors, but noted two caveats pertaining to their use within the diabetes domain [42]. First, more testing and development is required to understand efficacy [42]. Second, while patients are enthusiastic about virtual coaches, efforts on evaluating accuracy of responses is needed [42]. While our work represents the conceptual phase of DTEC, this notion of diabetes technology enabled coaching may be well positioned to address some of these concerns as a larger model of service tempered by human support. Within the context of DTEC, to more comprehensively address equity concerns, a larger model of service should be developed with community health workers or through partnerships with local organizations to amplify efforts of existing strategies to support scale and sustained use. This model of service could be designed to be agile and customizable for meeting unique community or individual needs. For example, DTEC could be deployed through a tiered approach offering support based on an individual’s digital and health literacy, or preference for communication style. We anticipate this ability to tailor DTEC would improve its sustained use at the individual level and address systemic barriers while offering a scalable solution more broadly. That said, further work is needed to continue exploring feasibility, sustainability, and the ability for DTEC to support behavior change, as well as evaluation of implementation and effectiveness of DTEC to more robustly inform scale and spread potential. Finally, as the REMIT program continues to build an evidence base for a multimodal remission strategy, the piloting and evaluation of DTEC to support remission evaluation is paramount to more robustly determine DTEC’s potential to equitably scale and spread REMIT.

### Recommendations to support implementation outcomes, scale and spread of DTEC

We offer the following recommendations for moving from the conceptualization phase of technology to support the design and development of diabetes health coaching while planning for successful implementation. We have summarized participant feedback (Table 1) through the cognitive walk-throughs to facilitate DTEC related to the primary objective implementation outcomes acceptability, adoption, feasibility, and appropriateness.

More broadly, considering feedback received by patients and health coaches, as well as our experiences with designing and developing digital health solutions, we propose three main recommendations for continued exploration of DTEC’s ability to support scale and spread.

1. Supporting access to resources: integration with existing devices (e.g., blood glucometers, fitness trackers) is essential to streamline data entry, particularly as diabetes remission approaches require intensive modification and tracking of health behaviors.
2. Supporting health knowledge disparities: Consideration for multiple methods for engagement (e.g., smart phone in addition to web application). The use of a chatbot may additionally support navigating available resources in a more natural way for users while potentially closing the gap on access if deployed through SMS and web services.
3. Supporting accessible design: human centered co-designing of DTEC and universal design principles [43] are necessary. We also stress the need for usability testing with a representative sample to ensure inadvertent violations are identified and corrected.

**Table 1:** Barriers and recommendations relating to acceptability, adoption, feasibility explored through cognitive walkthrough feedback.

IOF Element [44]	Identified Barriers	Recommendations
Acceptability (palatability)	• Contrast insufficient to be legible (low contrast)	• Implement Universal Design Principles
Adoption (intention to use)	• Limited to smart devices	• Web app as a complement
	• Format of presenting information may prohibit use (feel like a failure)	• Allow data visualization that is supportive of mental wellbeing, motivation, and how each individual views progress
	• Tracking only may not be motivating enough	• “I feel like the chances of a user actually adopting the app and using it on a regular basis it would be higher if you [had more interactive elements like personalized tips]”
Feasibility (extent to which can be successfully used)	• Access to diabetes hardware equipment may be cost prohibitive	• Integrate with existing bluetooth devices (e.g. fitness trackers, blood glucometer)
	• Data entry burden	• Provide alternatives to manual data entry (e.g., chatbot)
Appropriateness (perceived fit-for-purpose)	• Actionable feedback required to operationalise trends	• Expand data types to support varying lifestyles and life moments • Allow for flexibility of data entry
	• Educational components are desired	• Support messaging feature • Provide prepared education messages • Link to trusted diabetes resources

### Limitations and future directions

This study adds to existing literature on opportunities for technology to support T2D management. However, our relatively small sample size of patients and health coaches represents only a specific subgroup within the T2D population which may yield different outcomes compared to other subgroups (patient sample: 67% female, 50% advanced or professional degree). Given the equity considerations that came up within this work, as part of future pilots and trials, efforts to recruit a more diverse and representative sample is needed to ensure robust and representative voices and experiences are reflected. This includes the need to collect and analyze additional demographics to capture potential impacts not only of age, sex, gender, and education, but to explicitly probe important sociocultural and socioeconomic factors. While outside the scope of this foundational assessment for DTEC, further implementation of universal design principles [43], and the need for rigorous usability testing with a larger, representative sample is necessary to ensure inadvertent violations are identified and corrected as DTEC solutions are developed. Similarly, equitable access should be explicitly and formally evaluated in future usability works including piloting design concepts with a larger, diverse sample of patients. Additionally, determining essential training aspects for patients and healthcare providers, and privacy concerns is warranted to ensure implementation success, as well as a cost-effectiveness study to inform on sustainability. Continuing to probe these constructs will be crucial as the remission trial within and outside of the DTEC context is expanded to different populations. To further the learnings gathered here, work exploring nuances of barriers and facilitators to scaling and spreading DTEC would benefit future pilots and trials. Additionally, further research and pilot studies are needed to verify these findings and to explore additional implementation outcomes (e.g., sustainability, fidelity, cost), service outcomes, and client outcomes.

### Conclusion

Diabetes technology enabled coaching has the potential to address previous shortcomings of the multicomponent REMIT intervention. Semi-structured interviews with patients and health coaches suggest diabetes technology enabled coaching is primed for acceptability, adoption, feasibility, and appropriateness. Further opportunities to support diabetes technology enabled coaching include enhancing self-management with low-barrier communication and personalized education; however, several considerations are necessary to support equitable access to this approach. First, integration with existing devices is needed to streamline data entry. Second, consideration for technology provision, training, alternative program offerings are essential (e.g., web-based or SMS). Third, universal design principles should be consulted for accessible design. This is in addition to the need for human centered co-design with patients, health coaches and community partners and rigorous usability testing with a representative sample. Future work piloting DTEC in real-world settings, a formal evaluation of effectiveness, sustainability, scalability, factors required to support equity and to address privacy and safety are important next steps.

## Materials and Methods

This study was conducted at Hamilton Health Sciences (HHS) and the University Health Network (UHN). Data were collected from February 24^th^ to April 14^th^, 2022. Recruitment and data collection were conducted virtually. Written informed consent was obtained from all participants prior to interviews by the research team via the REDCap Platform (Research Electronic Data Capture) [45,46] hosted at UHN. Ethical approval was obtained both through the UHN and HHS Research Ethics Boards (Project ID: 20–6031.0 and HHS 14287).

### Study design

This was a qualitative descriptive study [47]. We leveraged human-centred design (HCD) to guide the co-design of DTEC. HCD is an evidence-based iterative process involving engaged users’ perspectives throughout the design process including from needs assessment and concept generation, to prototyping designs and system development, to evaluation of prototypes [48,49]. Research activities included baseline questionnaires, semi-structured interviews, and cognitive walkthroughs [50] of prototypes. Prototypes for a future DTEC were developed leveraging an existing application, *bant*. *Bant* is a behavioural mobile app for the self-management of diabetes which facilitates structured SMBG are key events, such as meals and physical activity, but does not currently offer health coaching or REMIT support [51].

#### Sampling

We engaged participants throughout the co-design process to ensure end-users’ needs and experiences guided the technical solution. Specifically, our sample of participants included patients living with T2D, and health coaches with previous experience with any REMIT study. More specifically, eligibility included (1) patients living with diagnosed T2D, or health coaches providing care for individuals with T2D, (2) having participated in a REMIT trial, (3) over the age of 18 years, and (4) understand English sufficiently to consent and complete study activities. We estimated a sample of three to six patients and three to six health coaches to reach thematic saturation.

#### Data collection

In alignment with qualitative principles of theoretical data saturation, our team iteratively assessed and reassessed the need for additional interviews throughout the data collection period to ensure conceptual depth was achieved [52]. Recruitment targets were met with a purposeful sample of 6 patients and 6 health coaches and conceptual depth was achieved with good overlap across participant feedback. Due to the COVID-19 pandemic, data collection was collected virtually through a secure video conferencing platform (Microsoft Teams). After obtaining informed written consent (REDCap) [45,46], all participants completed a testing session of 60–90 minutes inclusively consisting of a pre-study questionnaire (patients only), semi-structured interviews, and a cognitive walkthrough [50]. The patient-only pre-study questionnaire probed demographics, experience with diabetes management, and technology use. Semi-structured interviews were conducted with research staff. Interview guides (S1 Appendix) were designed to probe: (1) experiences with T2D management before and during the REMIT study, (2) participants’ level of comfort with using technology, and (3) participants’ level of comfort with integrating technology into the REMIT intervention. All participants completed a cognitive walkthrough to garner feedback on an early prototype to support the digitally-enabled version of REMIT. Each group (patients and health coaches) was presented with various scenarios, specific to their role, and shown DTEC prototypes from associated screens from the digital tool, *bant* (via the screen share option on Microsoft Teams).

#### Data analysis

Participant demographic data was analyzed via descriptive statistics. To contextualize our data, while ensuring high-quality qualitative analysis, we outlined our team’s positionality to describe how each of the researchers’ identities, values, experiences, and social positionings may have influenced data collection, analysis and interpretation [53] (S2 Appendix).

Transcription of interviews and cognitive walkthroughs were conducted by UHN research team members. Transcripts were analyzed in two phases. First, DN and MT developed a codebook based on emerging themes informed from thematic content analysis of the interview data using Nvivo software (Version 12). During codebook development, authors DN, MT reviewed four transcripts which was independently applied to two additional transcripts by DN and MT. Each coder independently coded assigned transcripts. From there, the codebook was iteratively refined to remove ambiguity and overlap while providing flexibility for adding emerging themes. The finalized codebook was used for formal analysis. The second phase of analysis, completed by MT, DN, and KJP, involved mapping themes onto concepts of acceptability, adoption, feasibility, which were loosely informed by the Implementation Outcome Framework (IOF) [44]. Finally, these IOF concepts were linked based on how they relate to the scale and spread of the REMIT intervention.

## Supporting information

S1 Appendix Patient and Health Coach Interview Guides. Caption: S1 Appendix provides a copy of the interview guides used for this work; the patient interview guide appears first followed by the health coach interview guide.
(PDF)

S2 Appendix Team Positionality Statement. Caption: S2 Appendix outlines our team’s positionality to describing identities, values, experiences, and social positionings may have influenced data collection, analysis and interpretation for each of the researchers involved in data analysis.
(PDF)

## References

[1] Lipscombe L, Booth G, Butalia S, Dasgupta K, Eurich DT, Goldenberg R, et al. Pharmacologic Glycemic Management of Type 2 Diabetes in Adults. Can J Diabetes. 2018;42: S88–S103. doi: 10.1016/j.jcjd.2017.10.034 29650116

[2] Brown JB, Nichols GA, Perry A. The burden of treatment failure in type 2 diabetes. Diabetes Care. 2004;27: 1535–1540. doi: 10.2337/diacare.27.7.1535 15220224

[3] Harris SB, Ekoé J-M, Zdanowicz Y, Webster-Bogaert S. Glycemic control and morbidity in the Canadian primary care setting (results of the diabetes in Canada evaluation study). Diabetes Res Clin Pract. 2005;70: 90–97. doi: 10.1016/j.diabres.2005.03.024 15890428

[4] Kankeu HT, Saksena P, Xu K, Evans DB. The financial burden from non-communicable diseases in low- and middle-income countries: a literature review. Health Res Policy Syst. 2013;11: 31. doi: 10.1186/1478-4505-11-31 23947294 PMC3751656

[5] Bailey CJ, Kodack M. Patient adherence to medication requirements for therapy of type 2 diabetes. Int J Clin Pract. 2011;65: 314–322. doi: 10.1111/j.1742-1241.2010.02544.x 21314869

[6] Wexler DJ, Grant RW, Wittenberg E, Bosch JL, Cagliero E, Delahanty L, et al. Correlates of health-related quality of life in type 2 diabetes. Diabetologia. 2006;49: 1489–1497. doi: 10.1007/s00125-006-0249-9 16752167

[7] Boocock RC, Lake AA, Haste A, Moore HJ. Clinicians’ perceived barriers and enablers to the dietary management of adults with type 2 diabetes in primary care: A systematic review. J Hum Nutr Diet. 2021;34: 1042–1052. doi: 10.1111/jhn.12875 33761151

[8] Buse JB, Caprio S, Cefalu WT, Ceriello A, Del Prato S, Inzucchi SE, et al. How do we define cure of diabetes? Diabetes Care. 2009;32: 2133–2135. doi: 10.2337/dc09-9036 19875608 PMC2768219

[9] Gong Q, Gregg EW, Wang J, An Y, Zhang P, Yang W, et al. Long-term effects of a randomised trial of a 6-year lifestyle intervention in impaired glucose tolerance on diabetes-related microvascular complications: the China Da Qing Diabetes Prevention Outcome Study. Diabetologia. 2011;54: 300–307. doi: 10.1007/s00125-010-1948-9 21046360

[10] Li G, Zhang P, Wang J, An Y, Gong Q, Gregg EW, et al. Cardiovascular mortality, all-cause mortality, and diabetes incidence after lifestyle intervention for people with impaired glucose tolerance in the Da Qing Diabetes Prevention Study: a 23-year follow-up study. The Lancet Diabetes & Endocrinology. 2014;2: 474–480. doi: 10.1016/S2213-8587(14)70057-9 24731674

[11] Jacob E, Avery A. Energy-restricted interventions are effective for the remission of newly diagnosed type 2 diabetes: A systematic review of the evidence base. Obes Sci Pract. 2021;7: 606–618. doi: 10.1002/osp4.504 34631138 PMC8488441

[12] Rehackova L, Araújo-Soares V, Steven S, Adamson AJ, Taylor R, Sniehotta FF. Behaviour change during dietary Type 2 diabetes remission: a longitudinal qualitative evaluation of an intervention using a very low energy diet. Diabet Med. 2020;37: 953–962. doi: 10.1111/dme.14066 31269276

[13] Goldenberg JZ, Johnston BC. Low and very low carbohydrate diets for diabetes remission. BMJ. 2021;373: n262. doi: 10.1136/bmj.n262 34031034

[14] Dixon JB, O’Brien PE, Playfair J, Chapman L, Schachter LM, Skinner S, et al. Adjustable gastric banding and conventional therapy for type 2 diabetes: a randomized controlled trial. JAMA. 2008;299: 316–323. doi: 10.1001/jama.299.3.316 18212316

[15] Mingrone G, Panunzi S, De Gaetano A, Guidone C, Iaconelli A, Leccesi L, et al. Bariatric surgery versus conventional medical therapy for type 2 diabetes. N Engl J Med. 2012;366: 1577–1585. doi: 10.1056/NEJMoa1200111 22449317

[16] Mingrone G, Panunzi S, De Gaetano A, Guidone C, Iaconelli A, Nanni G, et al. Bariatric–metabolic surgery versus conventional medical treatment in obese patients with type 2 diabetes: 5 year follow-up of an open-label, single-centre, randomised controlled trial. Lancet. 2015;386: 964–973. doi: 10.1016/S0140-6736(15)00075-6 26369473

[17] Gloy VL, Briel M, Bhatt DL, Kashyap SR, Schauer PR, Mingrone G, et al. Bariatric surgery versus non-surgical treatment for obesity: a systematic review and meta-analysis of randomised controlled trials. BMJ. 2013;347: f5934. doi: 10.1136/bmj.f5934 24149519 PMC3806364

[18] Wu G-Z, Cai B, Yu F, Fang Z, Fu X-L, Zhou H-S, et al. Meta-analysis of bariatric surgery versus non-surgical treatment for type 2 diabetes mellitus. Oncotarget. 2016;7: 87511–87522. doi: 10.18632/oncotarget.11961 27626180 PMC5350006

[19] Yan Y, Sha Y, Yao G, Wang S, Kong F, Liu H, et al. Roux-en-Y Gastric Bypass Versus Medical Treatment for Type 2 Diabetes Mellitus in Obese Patients: A Systematic Review and Meta-Analysis of Randomized Controlled Trials. Medicine. 2016;95: e3462. doi: 10.1097/MD.0000000000003462 27124041 PMC4998704

[20] Weng J, Li Y, Xu W, Shi L, Zhang Q, Zhu D, et al. Effect of intensive insulin therapy on beta-cell function and glycaemic control in patients with newly diagnosed type 2 diabetes: a multicentre randomised parallel-group trial. Lancet. 2008;371: 1753–1760. doi: 10.1016/S0140-6736(08)60762-X 18502299

[21] Riddle MC, Cefalu WT, Evans PH, Gerstein HC, Nauck MA, Oh WK, et al. Consensus Report: Definition and Interpretation of Remission in Type 2 Diabetes. Diabetes Care. 2021;44: 2438–2444. doi: 10.2337/dci21-0034 34462270 PMC8929179

[22] Chen H-S, Wu T-E, Jap T-S, Hsiao L-C, Lee S-H, Lin H-D. Beneficial effects of insulin on glycemic control and beta-cell function in newly diagnosed type 2 diabetes with severe hyperglycemia after short-term intensive insulin therapy. Diabetes Care. 2008;31: 1927–1932. doi: 10.2337/dc08-0075 18556343 PMC2551629

[23] Kramer CK, Zinman B, Retnakaran R. Short-term intensive insulin therapy in type 2 diabetes mellitus: a systematic review and meta-analysis. Lancet Diabetes Endocrinol. 2013;1: 28–34. doi: 10.1016/S2213-8587(13)70006-8 24622264

[24] Beck-Nielsen H, Pedersen O, Lindskov HO. Normalization of the insulin sensitivity and the cellular insulin binding during treatment of obese diabetics for one year. Acta Endocrinol. 1979;90: 103–112. doi: 10.1530/acta.0.0900103 760352

[25] Kelley DE, Kuller LH, McKolanis TM, Harper P, Mancino J, Kalhan S. Effects of moderate weight loss and orlistat on insulin resistance, regional adiposity, and fatty acids in type 2 diabetes. Diabetes Care. 2004;27: 33–40. doi: 10.2337/diacare.27.1.33 14693963

[26] Lim EL, Hollingsworth KG, Aribisala BS, Chen MJ, Mathers JC, Taylor R. Reversal of type 2 diabetes: normalisation of beta cell function in association with decreased pancreas and liver triacylglycerol. Diabetologia. 2011;54: 2506–2514. doi: 10.1007/s00125-011-2204-7 21656330 PMC3168743

[27] McInnes N, Smith A, Otto R, Vandermey J, Punthakee Z, Sherifali D, et al. Piloting a Remission Strategy in Type 2 Diabetes: Results of a Randomized Controlled Trial. J Clin Endocrinol Metab. 2017;102: 1596–1605. doi: 10.1210/jc.2016-3373 28324049

[28] McInnes N, Hall S, Sultan F, Aronson R, Hramiak I, Harris S, et al. Remission of Type 2 Diabetes Following a Short-term Intervention With Insulin Glargine, Metformin, and Dapagliflozin. J Clin Endocrinol Metab. 2020;105. doi: 10.1210/clinem/dgaa248 32403130

[29] McInnes N, Hall S, Hramiak I, Sigal RJ, Goldenberg R, Gupta N, et al. Remission of type 2 diabetes following a short-term intensive intervention with insulin glargine, sitagliptin, and metformin: Results of an open-label randomized parallel-design trial. Diabetes Care. 2022;45: 178–185. Available: https://diabetesjournals.org/care/article-abstract/45/1/178/139010 doi: 10.2337/dc21-0278 34728531

[30] McInnes N, Hall S, Lochnan HA, Harris SB, Punthakee Z, Sigal RJ, et al. Diabetes remission and relapse following an intensive metabolic intervention combining insulin glargine/lixisenatide, metformin and lifestyle approaches: Results of a randomised controlled trial. Diabetes Obes Metab. 2023. doi: 10.1111/dom.15234 37580972

[31] McInnes N, Hall S, Lochnan H, Harris S, Punthakee Z, Sigal R, et al. 677-P: Remission of type 2 diabetes following intensive treatment with insulin glargine, lixisenatide, metformin, and lifestyle approaches: Results of a multicenter randomized controlled trial. Diabetes. 2021. doi: 10.2337/db21-677-p

[32] Li Y, Xu W, Liao Z, Yao B, Chen X, Huang Z, et al. Induction of Long-term Glycemic Control in Newly Diagnosed Type 2 Diabetic Patients Is Associated With Improvement of β-Cell Function. Diabetes Care. 2004;27: 2597–2602. doi: 10.2337/diacare.27.11.2597 15504992

[33] Nelson LA, Greevy RA, Spieker A, Wallston KA, Elasy TA, Kripalani S, et al. Effects of a Tailored Text Messaging Intervention Among Diverse Adults With Type 2 Diabetes: Evidence From the 15-Month REACH Randomized Controlled Trial. Diabetes Care. 2021;44: 26–34. doi: 10.2337/dc20-0961 33154039 PMC7783936

[34] Alvarado MM, Kum H-C, Gonzalez Coronado K, Foster MJ, Ortega P, Lawley MA. Barriers to Remote Health Interventions for Type 2 Diabetes: A Systematic Review and Proposed Classification Scheme. J Med Internet Res. 2017;19: e28. doi: 10.2196/jmir.6382 28193598 PMC5329647

[35] Agarwal S, Simmonds I, Myers AK. The Use of Diabetes Technology to Address Inequity in Health Outcomes: Limitations and Opportunities. Curr Diab Rep. 2022;22: 275–281. doi: 10.1007/s11892-022-01470-3 35648277 PMC9157044

[36] Wlasak W, Zwanenburg SP, Paton C. Supporting autonomous motivation for physical activity with chatbots during the COVID-19 pandemic: Factorial experiment. JMIR Form Res. 2023;7: e38500. doi: 10.2196/38500 36512402 PMC9879319

[37] Aggarwal A, Tam CC, Wu D, Li X, Qiao S. Artificial intelligence-based chatbots for promoting health behavioral changes: Systematic review. J Med Internet Res. 2023;25: e40789. doi: 10.2196/40789 36826990 PMC10007007

[38] Dennis B, Schöbel S, Janson A, Leimeister JM. Engaging Minds–How Gamified Chatbots can Support and Motivate Learners in Digital Education. Social Science Research Network. 2024. Available: https://papers.ssrn.com/abstract=4677036

[39] Svensson I, Nordström T, Lindeblad E, Gustafson S, Björn M, Sand C, et al. Effects of assistive technology for students with reading and writing disabilities. Disabil Rehabil Assist Technol. 2021;16: 196–208. doi: 10.1080/17483107.2019.1646821 31418305

[40] Sriwisathiyakun K, Dhamanitayakul C. Enhancing digital literacy with an intelligent conversational agent for senior citizens in Thailand. Educ Inf Technol. 2022;27: 6251–6271. doi: 10.1007/s10639-021-10862-z 35002466 PMC8727474

[41] Gupta S, Chen Y. Supporting inclusive learning using chatbots? A chatbot-led interview study. J Inf Syst Educ. 2022;33: 98–108. Available: https://aisel.aisnet.org/jise/vol33/iss1/11

[42] Lyzwinski LN, Elgendi M, Menon C. Conversational agents and avatars for cardiometabolic risk factors and lifestyle-related behaviors: Scoping review. JMIR MHealth UHealth. 2023;11: e39649. doi: 10.2196/39649 37227765 PMC10251225

[43] The 7 Principles. In: Centre for Excellence in Universal Design [Internet]. [cited 29 Jan 2024]. Available: https://universaldesign.ie/about-universal-design/the-7-principles

[44] Proctor E, Silmere H, Raghavan R, Hovmand P, Aarons G, Bunger A, et al. Outcomes for implementation research: conceptual distinctions, measurement challenges, and research agenda. Adm Policy Ment Health. 2011;38: 65–76. doi: 10.1007/s10488-010-0319-7 20957426 PMC3068522

[45] Harris PA, Taylor R, Thielke R, Payne J, Gonzalez N, Conde JG. Research electronic data capture (REDCap)—a metadata-driven methodology and workflow process for providing translational research informatics support. J Biomed Inform. 2009;42: 377–381. doi: 10.1016/j.jbi.2008.08.010 18929686 PMC2700030

[46] Harris PA, Taylor R, Minor BL, Elliott V, Fernandez M, O’Neal L, et al. The REDCap consortium: Building an international community of software platform partners. J Biomed Inform. 2019;95: 103208. doi: 10.1016/j.jbi.2019.103208 31078660 PMC7254481

[47] Colorafi KJ, Evans B. Qualitative Descriptive Methods in Health Science Research. HERD. 2016;9: 16–25. doi: 10.1177/1937586715614171 26791375 PMC7586301

[48] Creswell JW, Poth CN. Qualitative Inquiry and Research Design: Choosing Among Five Approaches. SAGE Publications; 2016. Available: https://play.google.com/store/books/details?id=DLbBDQAAQBAJ

[49] McCurdie T, Taneva S, Casselman M, Yeung M, McDaniel C, Ho W, et al. mHealth consumer apps: the case for user-centered design. Biomed Instrum Technol. 2012;Suppl: 49–56. doi: 10.2345/0899-8205-46.s2.49 23039777

[50] Polson PG, Lewis C, Rieman J, Wharton C. Cognitive walkthroughs: a method for theory-based evaluation of user interfaces. Int J Man Mach Stud. 1992;36: 741–773. doi: 10.1016/0020-7373(92)90039-N

[51] Goyal S, Morita P, Lewis GF, Yu C, Seto E, Cafazzo JA. The Systematic Design of a Behavioural Mobile Health Application for the Self-Management of Type 2 Diabetes. Can J Diabetes. 2016;40: 95–104. doi: 10.1016/j.jcjd.2015.06.007 26455762

[52] Saunders B, Sim J, Kingstone T, Baker S, Waterfield J, Bartlam B, et al. Saturation in qualitative research: exploring its conceptualization and operationalization. Qual Quant. 2018;52: 1893–1907. doi: 10.1007/s11135-017-0574-8 29937585 PMC5993836

[53] Holmes. Researcher Positionality—A Consideration of Its Influence and Place in Qualitative Research—A New Researcher Guide. Shanlax International Journal of Education. Available: http://files.eric.ed.gov/fulltext/EJ1268044.pdf

