# Supplementary material for: The value of diabetes technology enabled coaching (DTEC) to support remission evaluation of medical interventions in T2D: Patient and health coach perspectives
Source: PLOS Digit Health. 2025 Jan 9;4(1):e0000701. doi: 10.1371/journal.pdig.0000701 (PMC11717255; doi:10.1371/journal.pdig.0000701)
Supplement: S1 Appendix — Caption: S1 Appendix provides a copy of the interview guides used for this work; the patient interview guide appears first followed by the health coach interview guide. (PDF) [file pdig.0000701.s001.pdf]

Interview Guide - Health Coach

Study Title
DTEC Usability Study: Assessing the Feasibility and Acceptability of Diabetes Technology Enabled Coaching (DTEC), to Support an Intensive Metabolic and Education Intervention for Individuals with Type 2 Diabetes

Principal Investigator
Joseph Cafazzo, PhD, PEng
Executive Director
Centre for Global eHealth Innovation
University Health Network
Joe.cafazzo@uhn.ca

Co-Investigator
Diana Sherifali, RN, PhD, CDE
Associate Professor
McMaster University School of Nursing
dsherif@mcmaster.ca

Study Coordinator
Madison Taylor
Research Analyst
Centre for Global eHealth Innovation
University Health Network
Madison.taylor2@uhn.ca
437-996-8998

Caitlin Nunn
Research Coordinator
Centre for Global eHealth Innovation
University Health Network
Caitlin.nunn@uhn.ca

Participant ID (to be completed if taking notes)- Date -

Introduction	The interview will be divided into two sections. In part 1 we will talk about your experience with your health coaching practice, the REMIT protocol and technology. In part 2 we will be addressing potential solutions for these problems and feasible applications in the type 2 diabetes remission programs. These questions are intended to help guide the conversation. However, because the goal is to understand your experiences, you should feel free to talk about anything you think might be important. You can also ask me to repeat or rephrase a question if you don't understand. Finally, you also have the choice to ask to skip a question or end the interview at any time.<br><br>The interview will be audio recorded to help with our analysis. The audio recordings will be transcribed (into a written record), which will be accessible by the research team. We are going to ask that you do not discuss any personal health information, or information that identifies you. This is done in an effort to protect your privacy, and the privacy of those around you. At the end you will be given a 25-dollar Amazon e-gift card.<br><br>Do you have any questions before we get started?<br><br>Final check before starting:<br>☐ MS Teams: Ensure Audio Recording is prepared/ready to go<br>☐ Inform them when you start recording
INTERVIEW<br>Theme 1:<br>Current practice and Diabetes Management	*Today is [insert date] and this interview is with [insert participant ID].*<br><br>This first set of questions is about your current diabetes management practice:<br><br>1. What is your clinical background?<br>2. How long have you been working as a diabetes health coach?<br>3. How would you describe the T2D patient population that you currently serve?<br>a. Demographic characteristics?<br>b. How many patients do you have under the health coaching arm of your practice?
Theme 2:<br><br>Comfort in integrating bant technology into remit	Next, we have some questions regarding integrating technology in the REMIT protocol.<br><br>1. Have you had any previous experience with virtual care?<br>2. How do you feel about performing health coaching virtually?<br>3. In what ways do you think technology would improve or not improve the REMIT protocol?<br>a. Where would you like to see increased efficiencies?<br>4. Are there any challenges or opportunities with the REMIT protocol you anticipate going forward?
USABILITY WALKTHROUGH	☐ Share screen<br>☐ Have the prototype open with the use cases<br>☐ Inform that you are sharing your screen and have them confirm they can see it

	Here is some background information on our project: We are trying to better understand how a digital platform could be integrated into diabetes remission protocols. To do this, patients living with diabetes would download the *bant* app and log their data such as blood glucose levels, food, exercise, and weight etc. The health coach would be able to see the data that the patient inputs into the app.<br><br>This platform would have two interfaces, one for people who live with T2D, and one for health coaches. Health coaches and people living with diabetes would be able to see their diabetes-related health data all in one place, and be able to track trends and changes over time.
Scenario 1:<br>Data Review During a Patient Appointment	You have logged into the *bant* clinician dashboard. Your first patient of the day has been in the study for the past 6 months and has been tracking their behaviours and glucose levels. You would like to discuss blood glucose, meal intake, weight, activity, labs, medication and overall progress with the patient.<br><br>Task 1: Patient Summary Review<br><br>Question A<br>How do you feel about the information presented in the **Patient Summary** page for conducting your clinical assessment?<br><br>Question B<br>How do you feel about using this dashboard to enter structured goals for your patient to see in their app?<br><br>Task 2: Blood Glucose Review<br>You want to review the patient's blood glucose levels over the past month and during the most recent week. Based on their values, you ask the patient to change their target blood glucose range.<br><br>Question A<br>How do you feel about the information presented in the **Blood Glucose** page for conducting your clinical assessment?<br><br>Task 3: Meal Intake Review<br><br>Question A<br>How do you feel about the information presented on the **Meal Intake** page for conducting your clinical assessment?<br><br>Question B<br>How useful is displaying patient's before- and after-meal photos for you?<br><br>Task 4: Weight Review<br><br>Question A<br>How do you feel about the information presented on the **Weight Tracking** page for conducting your clinical assessment?

	Task 5: Activity Review<br><br>Question A<br>How do you feel about the information presented on the **Activity Tracking** page for conducting your clinical assessment?<br><br><br>Task 6: Lab Review<br>Review the patient's most recent **HbA1c lab** results and discuss findings with patient<br><br>Question A<br>How do you feel about the information presented on the **Lab Results** page for conducting your clinical assessment?<br><br><br>Task 7: Medication Review<br>Question A<br>How do you feel about the information presented on the **Medication** page for conducting medication review and titration?<br><br>Question B<br>Would you be willing to manually enter medication information into the dashboard in order to adjust meds for the patient?<br><br>Question C<br>How important is it for you to see the medication adjustments in the clinician dashboard?<br><br><br>If interviewee requires clarification, the following prompts may be used:<br>• What are you able to gather on the patient?<br>• Is there anything that you like or dislike?<br>• Anything missing that would have been useful for you?<br>• Based on what you see, how would you expect to interact with this page?<br>• For pages that have drop-down menu for time range filtering, what time ranges would you find useful?
Scenario 2: Asynchronous Interactions with a Patient	After your appointment with the patient, you receive messages from the patient regarding their diabetes self-management.<br><br>Screen 1: *Banter* Messenger (Clinician Interface)<br><br>Question A<br>How do you feel about interacting asynchronously with your patients in between your appointments using a messaging application?<br><br>Question B<br>What functionalities are important to you if you're interacting with the patient through a messaging platform? Ie. is there anything that you like, dislike, or anything that you think is missing from this screen?

	Question C<br>If you do not like the idea of a messaging interface with your patients, what form (if any) of patient interaction would you prefer in between your appointments?
Scenario 3: Offboarding & Sustainable Diabetes Self-Management	Your patient has now completed the Remission study and will be discharged from the study. You are due for a follow up call with the patient to review how their blood glucose has been over the past month. They entered a month's worth of blood glucose readings, all from various times of the day (pre/post meals) and after activity. Based on the findings you want to discuss with the patient how they can continue to improve their diabetes self-management by using the *bant* app.<br><br>Question A<br>Upon completion of the Remission study, would you be interested in having a summary report outlining your patient's progress? Why or why not? If you do want a summary report, where would you store this information?<br><br>Question B<br>What would be meaningful information in the summary report? (i.e. what would you like to see?)<br><br>Question C<br>Would features or functions do you think would encourage your patients to continue to use the *bant app* to sustain healthy diabetes self-management behaviors after the study?
Usability Walkthrough Debrief Questions	1. How did you feel about the clinician dashboard in these scenarios?<br>2. Is there any other health related data that you would like to see presented to you for your patients?<br>3. Do you feel the bant dashboard would be useful for you to provide care to your patients during the Remission study? Why or Why not?
Conclusion	That's it for my questions, is there anything we didn't discuss about your experience with technology or the REMIT program that you wanted to discuss?<br><br>☐ Inform that you are turning off recording<br>☐ Ensure that recording has stopped<br>☐ Save recording<br><br>Thank you for your time today. This was very helpful and thank you for sharing your experience with me today, we really appreciate it. To show our appreciation, we will be emailing you a $25-dollar Amazon e-gift card.<br><br>In terms of next steps, we will analyze the feedback from the people living with T2D and health coaches in order to inform our designs for the mobile health tool for diabetes management.<br><br>Please feel free to contact us by email or phone if you have any questions or if you'd like to be updated on the status of the research and receive a summary of our findings once the study is complete. Our contact information is located on your consent form.

Notes	
