# Supplementary material for: The value of diabetes technology enabled coaching (DTEC) to support remission evaluation of medical interventions in T2D: Patient and health coach perspectives
Source: PLOS Digit Health. 2025 Jan 9;4(1):e0000701. doi: 10.1371/journal.pdig.0000701 (PMC11717255; doi:10.1371/journal.pdig.0000701)
Supplement: S2 Appendix — Caption: S2 Appendix outlines our team’s positionality to describing identities, values, experiences, and social positionings may have influenced data collection, analysis and interpretation for each of the researchers involved in data analysis. (PDF) [file pdig.0000701.s002.pdf]

S2 Appendix.

Team Positionality Statement

Author MT, a settler of European descent, was born, raised, and currently resides on the lands now known as the Greater Toronto Area, on Turtle Island. As a middle-class public health and health systems researcher, she has extensively interacted with the Ontario healthcare system in addition to experience in personal life both as a caregiver and as someone seeking treatment. She has some family members living with T2D although has not had direct involvement in support of their care. These experiences have deeply informed her empathy, guiding her approach when conducting interviews. To our participants: please rest assured that your stories are held in the utmost care.

Author DN acknowledges her standpoint as an upper-class, cis-gender woman of colour living in a large metropolitan area in Canada. She is a public health and health systems researcher, with a focus on patient experience and mobile health application adoption. She is a digital native with an excellent grasp of current technological trends. Prior to this study, she has not had direct interactions with individuals living with T2D and therefore cannot fully comprehend the breadth and depth of experiences of participants. DN conducted interviews and completed the thematic analysis.

Author KJP acknowledges her standpoint as a middle class, neurodiverse, non-binary, white female. KJP leverages multidisciplinary training in biomedical sciences, health studies, and systems design engineering with a focus on responsible artificial intelligence driven health services, and multimethod evaluation of these services. KJP has family with, but no direct lived experience with T2D and as such, cannot completely comprehend the experiences of participants. KJP did not complete interviews during the data collection phase, and is only familiar with study participants through the transcripts of participant interviews.
